# A Trispecific Anti-HIV Chimeric Antigen Receptor Containing the CCR5 N-Terminal Region

**DOI:** 10.3389/fcimb.2020.00242

**Published:** 2020-05-25

**Authors:** Agnes Hajduczki, David T. Danielson, David S. Elias, Virgilio Bundoc, Aaron W. Scanlan, Edward A. Berger

**Affiliations:** Laboratory of Viral Diseases, National Institute of Allergy and Infectious Diseases, National Institutes of Health, Bethesda, MD, United States

**Keywords:** HIV, HIV functional cure, immunotherapy, cell therapy, chimeric antigen receptor, CD4, mannose binding lectin, CCR5

## Abstract

Anti-HIV chimeric antigen receptors (CARs) promote direct killing of infected cells, thus offering a therapeutic approach aimed at durable suppression of infection emerging from viral reservoirs. CD4-based CARs represent a favored option, since they target the essential conserved primary receptor binding site on the HIV envelope glycoprotein (Env). We have previously shown that adding a second Env-binding moiety, such as the carbohydrate recognition domain of human mannose-binding lectin (MBL) that recognizes the highly conserved oligomannose patch on gp120, increases CAR potency in an *in vitro* HIV suppression assay; moreover it reduces the undesired capacity for the CD4 of the CAR molecule to act as an entry receptor, thereby rendering CAR-expressing CD8^+^ T cells susceptible to infection. Here, we further improve the bispecific CD4-MBL CAR by adding a third targeting moiety against a distinct conserved Env determinant, i.e. a polypeptide sequence derived from the N-terminus of the HIV coreceptor CCR5. The trispecific CD4-MBL-R5Nt CAR displays enhanced *in vitro* anti-HIV potency compared to the CD4-MBL CAR, as well as undetectable HIV entry receptor activity. The high anti-HIV potency of the CD4-MBL-R5Nt CAR, coupled with its all-human composition and absence of immunogenic variable regions associated with antibody-based CARs, offer promise for the trispecific construct in therapeutic approaches seeking durable drug-free HIV remission.

## Introduction

The development of antiretroviral therapy (ART) drug regimens represents an exceptional medical achievement that has enabled individuals infected with human immunodeficiency virus (HIV) to remain asymptomatic and lead essentially normal lives (Saag et al., 2018). However, drug toxicities, high costs, adherence difficulties, emergence of viral resistance, and challenges to medication accessibility to communities at greatest need represent major burdens for the global HIV population (Mouton et al., 2016). These factors are fueling extensive efforts to develop HIV curative approaches that either eradicate the infection from the body (“sterilizing cure”) or achieve long-term remission by durably suppressing the virus (“functional cure”) (Siliciano and Siliciano, 2016; Davenport et al., 2019; Ndung'u et al., 2019; Peterson and Kiem, 2019). Amongst the diverse HIV cure concepts under development, enhancement/engineering of HIV-specific T cell function is receiving considerable attention (Patel et al., 2016; Yang et al., 2018). A rare subset of individuals, termed “elite controllers,” are able to naturally maintain virus loads below the level of detection in the absence of ART, thanks in large part to their strong HIV-specific CD8+ T cell responses (Goulder and Deeks, 2018). This has fueled considerable interest in devising approaches to endow HIV-infected subjects with elite-controller-like T cell responses to enable long-term discontinuation of ART without a viral rebound. In this context of durable remission, anti-HIV chimeric antigen receptors (CARs) represent a particularly active area of research (Kuhlmann et al., 2018; Wagner, 2018; Kim et al., 2019; Liu et al., 2019; Mylvaganam et al., 2019).

CAR technology involves the design of synthetic protein constructs containing a recognition/targeting domain attached via a hinge and transmembrane elements to functional intracellular domains, typically sequences involved in T cell costimulation and activation (Srivastava and Riddell, 2015; June and Sadelain, 2018). When expressed on the T cell surface, the CARs mediate selective killing of the chosen target cell type. In the case of anti-HIV CARs, killing is based on recognition of the intact HIV envelope glycoprotein (Env) on the surface of infected cells. Env is a trimer of gp120/gp41 heterodimers produced by proteolytic cleavage of the gp160 precursor (Sanders and Moore, 2017; Ward and Wilson, 2017; Chen, 2019). Upon interaction with the primary receptor CD4 at the T cell surface, gp120 undergoes a conformational change that exposes the otherwise concealed/unformed binding site for coreceptor (CCR5 or CXCR4). Coreceptor binding in turn triggers further conformation changes leading to exposure of the gp41 subunit and insertion of its hydrophobic fusion peptide into the target cell membrane. The resulting fusion of the viral membrane with the plasma membrane culminates in HIV entry. The bulky surface-exposed gp120 subunit contains highly variable regions that aid the virus in immune evasion, as well as conserved elements involved in critical functions that can be targeted by therapeutic approaches.

From the earliest days (Bitton et al., 2001) until the present (Kuhlmann et al., 2018; Wagner, 2018; Kim et al., 2019; Liu et al., 2019; Mylvaganam et al., 2019), CD4 has been a favored component for the targeting domain of anti-HIV CARs, because it recognizes an essential conserved feature of gp120 that presumably must be retained for viral persistence and pathogenicity. Our group has reported improved bispecific designs of CD4-based CARs containing a second moiety that targets a distinct conserved determinant on gp120 (Liu et al., 2015; Ghanem et al., 2018). The purpose of the second moiety is to enhance the anti-HIV potency of the CAR, and to inhibit the undesired potential for the CD4 to act as an HIV entry receptor on the CAR-expressing T cell. Importantly, to minimize immunogenicity, both moieties derive from human protein sequences. The most promising construct employed the carbohydrate recognition domain of human mannose-binding lectin (MBL), which recognizes the highly conserved oligomannose patch on gp120 (Ghanem et al., 2018).

In contemplating additional motifs for further CAR enhancement, we considered the N-terminal region of the HIV coreceptor CCR5. This region binds to a highly conserved site on HIV-1 gp120, and has been shown to function in various structural contexts including synthetic peptides and recombinant proteins (Choe and Farzan, 2009; Gardner and Farzan, 2017). As a “self” sequence, the CCR5 amino terminal region is likely to be minimally immunogenic as a CAR component. Because the CCR5/gp120 interaction is highly CD4-dependent (Ward and Wilson, 2017; Chen, 2019), we studied its functionality in the context of CD4-based CAR constructs. The data presented herein describe potency and protective enhancements provided by the CCR5 N-terminal region, and suggest its potential value as a component of a trispecific anti-HIV CAR.

## Results

### CAR Constructs

**Figure 1:**
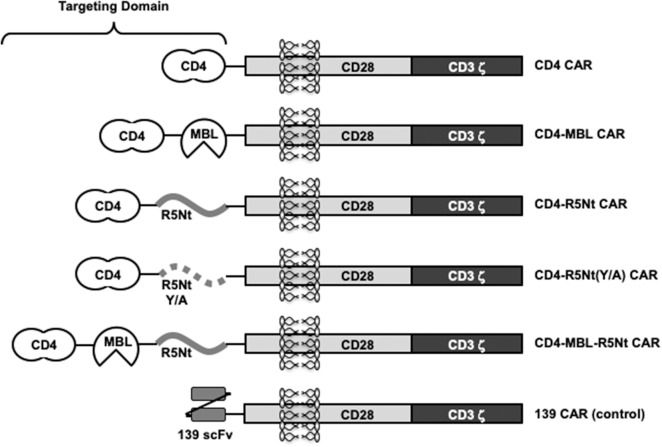
Schematic representation of various CAR constructs used in this study. “CD4” moiety is domains 1 and 2 of human CD4, residues 1-208, including the 25 residue signal peptide. “MBL” is the carbohydrate recognition domain of human mannose-binding lectin (residues 214-330). “R5Nt” represents the N-terminal segment (residues 2-26) of human CCR5, with a Cys to Ser substitution at residue 20. “R5Nt(Y/A)” is a control version of R5Nt containing four tyrosine-to-alanine substitutions that eliminate the sulfation targets required for binding to gp120. The recognition domain of the control 139 CAR is an irrelevant scFV. Solid black lines indicate flexible linkers of various lengths. See Supplementary Figure 1 for amino acid sequences.

Figure 1 shows the design of the CAR constructs analyzed in this report. In each case, the indicated targeting domain is attached via a 3 amino acid linker to a segment of human CD28 (truncated extracellular, transmembrane, and intracellular costimulatory domains) followed by the intracellular activation domain of human CD3 zeta. Within each targeting domain, the different recognition moieties are separated by linkers of 5 or 10 amino acids. The full amino acid sequences are shown in Supplementary Figure 1.

The monospecific CD4 CAR, the bispecific CD4-MBL CAR, and 139 control CAR (irrelevant scFv) have been reported previously (Liu et al., 2015; Ghanem et al., 2018). The new constructs contain a 25 amino acid segment based on residues 2-26 of human CCR5 (herein designated R5Nt). In keeping with previously reports using synthetic peptides (Farzan et al., 2000, 2002), residue 20 was changed from Cys to Ser to avoid inappropriate disulfide formation. We first generated a bispecific CAR designated CD4-R5Nt, *in order to evaluate R5Nt's potential as CAR component*, even when not located at the N-terminus of the recombinant protein. The native CCR5 N-terminus contains between 2 and 4 sulfated tyrosine residues at positions 2, 10, 14, 15, which have been shown to be necessary for HIV coreceptor function (Choe and Farzan, 2009). Structural studies indicate that it is the sulfate groups themselves, rather than the polypeptide side chains, that mediate the interaction with gp120 (Huang et al., 2007); in fact, the unsulfated CCR5 amino terminal region is inactive. Therefore, we constructed a control CAR, designated CD4-R5Nt(Y/A), wherein the four tyrosine residues were mutated to alanine.

### Expression and Env-Mediated Activation of the CD4-R5Nt CAR

**Figure 2:**
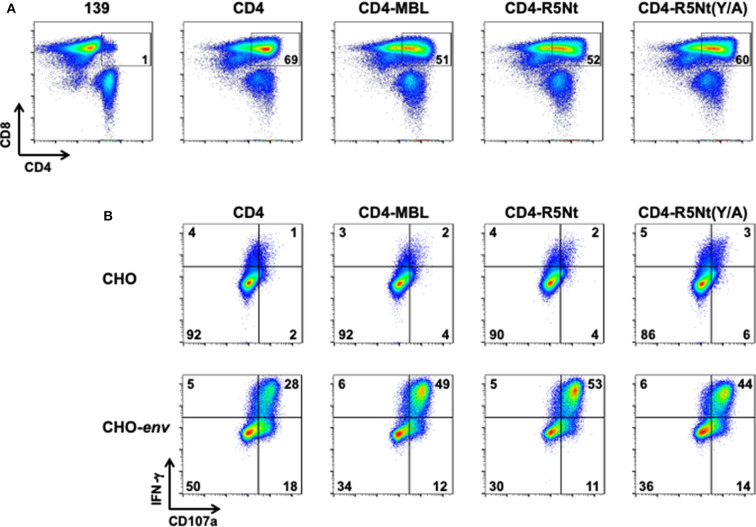
Flow cytometry analysis of surface expression and activation of CARs, analyzed at day 6 following PBMC transduction. **(A)** CAR surface expression. After gating on live T lymphocytes, CAR expression levels were determined by the presence of CD4 on the CD8^+^ T cell populations; inside boxes indicate % CD4-positive. **(B)** CAR activation by Env-expressing cells. CAR-transduced PBMCs were co-cultured for 6 h with either CHO cells (top), or CHO-env cells (bottom), which express surface HIV-1 Env. Cells were then stained for activation markers IFN–γ and CD107a. The % of cells in each quadrant are indicated.

Stimulated PBMCs from normal donor blood were transduced with retroviral vectors encoding the previously described CD4 and CD4-MBL CARs, as well at the CD4-R5Nt and CD4-R5Nt(Y/A) CARs. Figure 2A shows analysis of CAR surface expression by flow cytometry. Based on detection of surface CD4 on the CD8^+^ T cell population, CAR expression was found to be comparably high for all the CD4-based constructs (~50–70% CD4-positive, compared to ~1% for control cells transduced with the 139 CAR). We next tested whether the CAR-T cells can be activated by cells expressing surface HIV-1 Env. T cells expressing the various CD4-based CARs were co-cultured for 6 h with either parental CHO cells or CHO-*env* transfectant cells that stably express surface Env; brefeldin A and monensin were included in the co-cultures to enable staining for accumulated intracellular IFN-γ and production of the CD107a degranulation marker. The results shown in Figure 2B demonstrate minimal background activation mediated by any of the CARs upon co-culture with CHO cells, but dramatic upregulation of IFN-γ and CD107a in all the CD4-based CAR-T cells upon co-culture with CHO-*env* cells. The antigen-specific activation was somewhat greater with the bispecific CARs compared to the monospecific CD4 CAR.

### Effects of the R5Nt Moiety on Anti-HIV Activity in the Context of a Bispecific CD4-Based CAR

**Figure 3:**
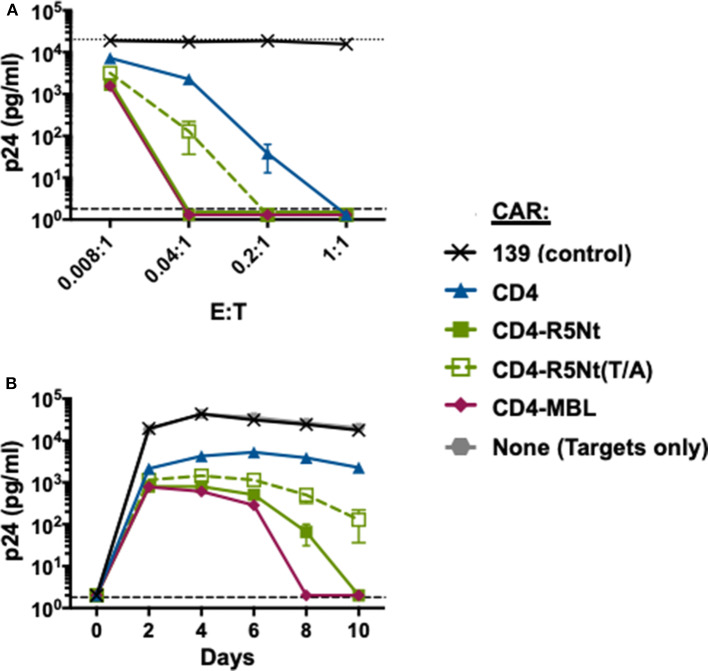
Effects of the R5Nt moiety in the context of bispecific CD4-based CARs. The activities of the various CARs were tested in the HIV-1 spreading infection assay (Ba-L primary isolate). PBMCs were transduced with retroviral vectors and cultured for 6 days to generate T cells expressing the indicated CARs (Effectors). Cocultures were performed with autologous PBMCs infected with HIV-1 (Targets). Cocultures at varying E:T ratios were continued for 10 days, with aliquots taken at 2-day intervals for p24 assay. **(A)** Effects of varying E:T ratios. p24 values are shown for samples collected on Day 10. The dotted line represents Target cells only. **(B)** Time course of infection. p24 values are shown for cocultures at E:T ratio of 0.04:1. In both graphs, the dashed line represents the limit of detection determined from the standard curve. All assay points represent the mean of triplicate samples, with error bars denoting standard deviation.

To assess the anti-HIV activities of the CARs, we performed *in vitro* spreading infection coculture assays as described previously (Liu et al., 2015; Ghanem et al., 2018). PBMC from the same donor were infected with HIV-1 and incubated overnight to generate “target” cells. The following day, cocultures were established containing a fixed number of infected target cells plus CAR-expressing “effector” cells, at various effector-to-target (E:T) ratios (ranging from 0.008:1 to 1:1). Controls included cultures with no effector cells, or with effectors transduced with the irrelevant 139 control CAR. At 2-day intervals, aliquots of supernatants were collected for analysis of p24 content. Results with the HIV-1 primary isolate BX08 isolate are shown in Figure 3. As one form of analysis, CAR potencies were compared at varying E:T ratios (Figure 3 Top, day 10). At the highest E:T ratio of 1:1, all CD4-containing CARs gave full suppression, with p24 levels below detectable limits. However, significant potency differences were revealed at lower E:T ratios. The bispecific CD4-R5Nt CAR, like the previously described CD4-MBL CAR (Ghanem et al., 2018), displayed higher potency than the monospecific CD4 CAR. A similar pattern emerged from analysis CAR activities over the time course of infection (Figure 3 Bottom, E:T of 0.04:1); the bispecific CD4-R5Nt CAR was significantly more potent than the CD4 monospecific CAR, approaching the efficacy of the CD4-MBL CAR. In both the varying E:T ratio and the time course analyses, the CD4-R5Nt was more potent than the mutant CD4-R5Nt(Y/A) CAR, presumably reflecting specific interaction of the CCR5 N-terminal moiety with its cognate coreceptor binding site on HIV-1 gp120. The mutant CD4-R5Nt(Y/A) CAR also displayed somewhat higher potency than the CD4 CAR, indicative of effects unrelated to specific binding.

### Expression and Anti-HIV Activity of a Trispecific CD4-Based CAR Containing the R5Nt Moiety

**Figure 4:**
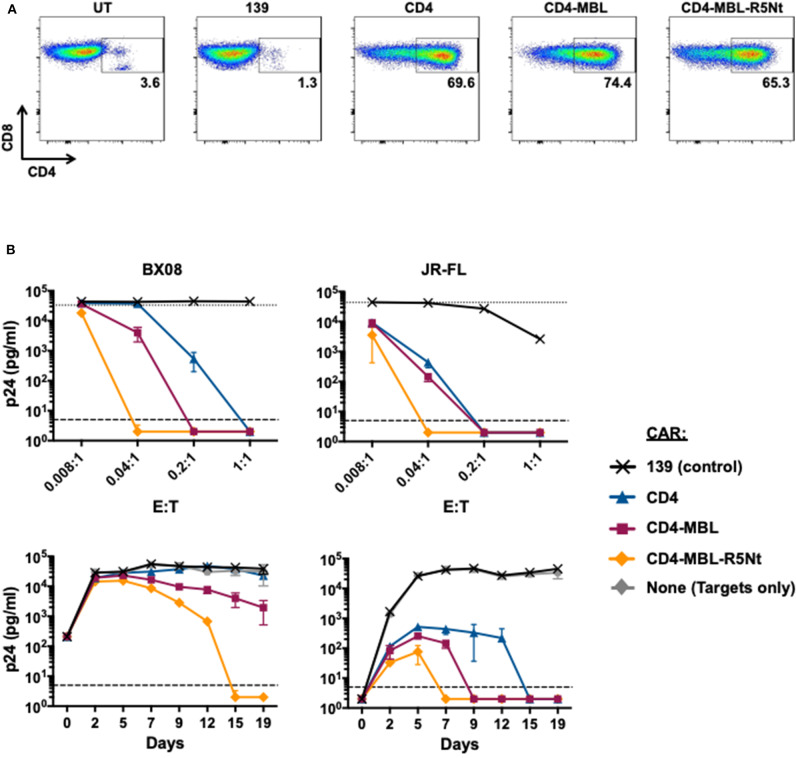
Effects of the R5Nt moiety in the context of the trispecific CD4-MBL-R5Nt CAR. **(A)** Flow cytometry analysis of surface expression of CARs. After gating on live T lymphocytes, the levels of CAR expression were determined by the presence of CD4 on the CD8^+^ T cell populations (inside box). Cells were analyzed at day 11 post-transduction; inside boxes indicate % CD4-positive. **(B)** Activities of CARs in the HIV-1 spreading infection assay for the BX08 (left panels) and JR-FL (right panels) primary isolates. Cocultures at varying E:T ratios were continued for 19 days, with aliquots taken at 2-3-day intervals for p24 assay. Top panels show the effects of varying E:T ratios. p24 values are shown for BX08 at day 15, and for JR-Fl at day 7. In both graphs, the dotted line represents Target cells only. Bottom panels show the time courses of infection. P24 values are shown for cocultures at E:T ratio of 0.04:1. In all graphs, the dashed line represents the limit of detection determined from the standard curve. All assay points represent the mean of triplicate samples, with error bars denoting standard deviation.

The efficacy of the R5Nt moiety in the context of the bispecific CAR prompted us to analyze it's potential to enhance our previously favored CD4-MBL CAR construct. The trispecific CAR, herein referred to as CD4-MBL-R5Nt, showed high-level expression comparable to the other CD4-based CARs (Figure 4A). Anti-HIV-1 activities were analyzed in spreading infection assays. Figure 4B shows results with the primary HIV-1 isolates BX08 (left panels) and JR-FL (right panels). When analyzed in the varying E:T ratio mode (top panels) or the time course of infection (bottom panels), the CD4-MBL-R5Nt CAR displayed consistently higher potency than the CD4-MBL CAR.

### Full Blockage of CAR-Mediated HIV Entry Receptor Activity in the Trispecific CAR

**Figure 5:**
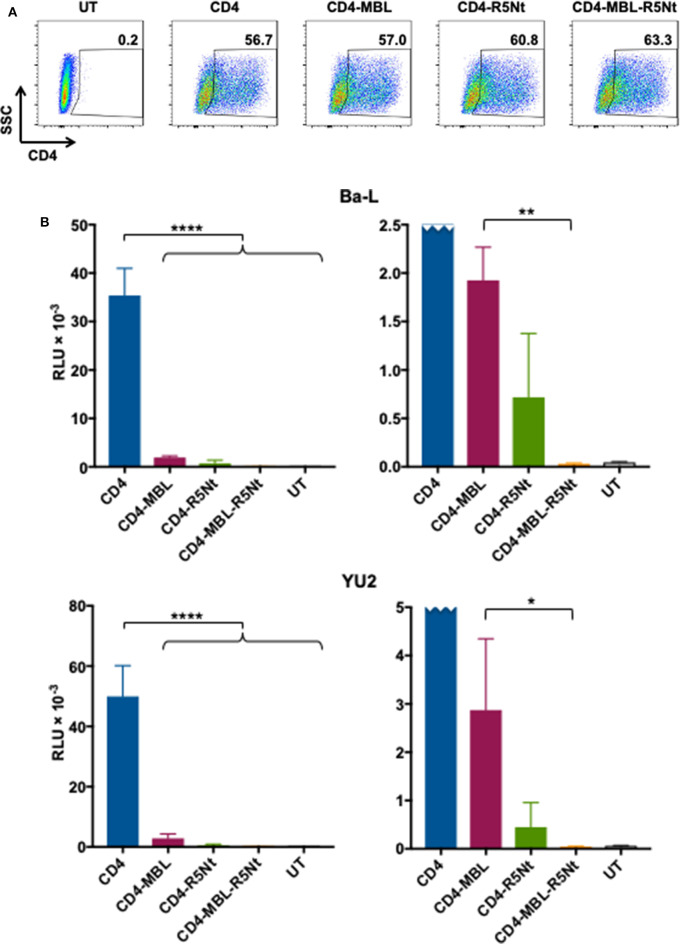
Ability of the CD4 moiety of various CAR molecules to function as an entry receptor for HIV-1 pseudoviruses. **(A)** Flow cytometry analysis of CAR expression on transfected HOS.CCR5 cells. Following gating on live cells, CAR expression was determined based on surface CD4 levels; inside boxes indicate % CD4-positive. **(B)** Pseudovirus entry receptor activity. HOS.CCR5 cells transfected to express the indicated CAR (or untransfected, UT) were analyzed in the HIV-1 pseudovirus entry assay using pseudoviruses bearing Envs from the Ba-L (top) or YU2 (bottom) primary isolates. Pseudovirus entry was determined at 48 h based on luciferase activity. Assay points represent the mean of quadruplicate samples, with error bars denoting standard deviation. For the left panel (full scale), statistics was performed by one-way ANOVA, comparing the CD4 CAR with each of the multispecific CARs; ****P < 0.0001. For the right panel (expanded scale, CD4 CAR off-scale), statistics were performed by the paired *t*-test to compare the CD4-MBL bispecific CAR to the CD4-MBL-R5Nt trispecific CAR; **P = 0.0017, *P = 0.0313.

An important concern regarding CD4-based CARs is the potential for the CD4 moiety to act as an HIV entry receptor. This is particularly true for CD8^+^ CAR-T cells, which are presumed to be important effectors for CAR-mediated control. The presence of endogenous CCR5 on CD8^+^ T cells (Brenchley et al., 2004) makes these otherwise HIV-refractory cell types potentially vulnerable to infection via the transduced CD4 moiety, thus compromising their function and viability. We previously reported that the second moiety of a bispecific CAR, whether a single chain antibody variable fragment (Liu et al., 2015) or a carbohydrate recognition domain of a C-type lectin (Ghanem et al., 2018), significantly inhibits this undesired activity. However, some residual activity persists, as shown in the experiment detailed in Figure 5. We analyzed whether expression of the CD4-based CARs confers HIV-1 pseudovirus entry susceptibility to HOS.CCR5, a transfectant cell line stably expressing CCR5 (but not CD4). Figure 5A demonstrates comparable surface expression of the various CARs upon plasmid transfection of these cells. The pseudovirus entry assays shown in Figure 5B (left panels), using pseudoviruses from both primary strains Ba-L (upper panel) and YU2 (lower panel), demonstrate undetectable entry in untransfected HOS.CCR5 cells, but robust entry in cells transfected with the CD4 CAR. The bispecific CD4-MBL CAR conferred much lower entry permissiveness for both strains, as did the bispecific CD4-R5Nt CAR, indicating that the second moiety of each construct significantly impaired the capacity of the CD4 to serve as an entry receptor. However, for both bispecific CARs, low levels of pseudovirus entry were in fact detected, as best illustrated in the expanded graphs (right panels). Here, the superiority of the trispecific CD4-MBL-R5Nt CAR is clearly revealed, with significantly lower entry activities compared to the CD4-MBL CAR, for pseudovirus from both the Ba-L and YU2 strains; in fact, entry mediated by the trispecific CAR is indistinguishable from the background observed in untransfected cells.

## Discussion

CAR technology has potential to promote durable suppression of HIV in the absence of antiretroviral drugs. Because of the lifelong persistence of latently infected cellular reservoirs (Sengupta and Siliciano, 2018), an HIV “functional cure” would presumably require long-term (life-long?) persistence and functional efficacy of the CAR-T cells. To achieve these ends, we believe that anti-HIV CAR design must strive not only to optimize potency, but also to minimize both immunogenicity and potential for viral mutational escape. CD4 thus seems an ideal targeting component: as a “self” protein it is predicted to have low immunogenicity, and as the primary HIV receptor, it presumably cannot be escaped without major losses to viral fitness and pathogenicity. Strict killing specificity for Env-expressing cells stems from previous findings that CD4-based CARs do not recognize or kill cells expressing surface MHC class II (Romeo and Seed, 1991; Liu et al., 2015; Leibman et al., 2017), presumably due to the very weak binding affinities of these molecules, as previously discussed (Ghanem et al., 2018). Our approach has involved design of multispecific CD4-based CARs, with the additional component(s) intended to both enhance anti-HIV potency and minimize entry receptor activity of the CD4 moiety. While we initially achieved success with an scFv against the coreceptor binding site as the second moiety (Liu et al., 2015), we have since diverged from antibody-based motifs because of immunogenicity concerns of the associated variable regions. Indeed, efficacy-impairing anti-idiotypic antibody responses have been reported for therapeutic antibodies (Krishna and Nadler, 2016; Martinez-Navio et al., 2016) and scFv-based CARs (Lamers et al., 2011). Hence our shift to non-antibody “self” sequences, such as the previously reported carbohydrate recognition domain of human MBL (Ghanem et al., 2018), and the present use of the CCR5 amino terminal region.

The greater potency of the bispecific CD4-R5Nt CAR relative to the CD4-R5Nt(Y/A) variant (Figure 3) speaks to the enhancement associated with specific binding of the second moiety to its cognate site on gp120. However, non-specific effects also occurred, as indicated by the modestly increased potency of the CD4-R5Nt(Y/A) variant compared to the monospecific CD4 CAR (Figure 3). Multiple factors may contribute to enhancement conferred by the non-binding mutant moiety, including increased CD4 distance from the effector cell surface, changes in chain flexibility, and other variables that might influence access/binding of the CD4 moiety to gp120 on the target cell surface.

In considering possible mechanisms governing the enhanced potency of the multispecific CARs, we note that increased binding affinity for gp120 is an unlikely explanation. The linkers between the different recognition components are only 5 or 10 amino acid residues, far too short to enable multiple moieties of a single CAR molecule to simultaneously engage their cognate binding sites on a single gp120 subunit. In fact, our previous bispecific CAR studies demonstrated that potency was actually enhanced by deliberately rendering the linker between the two recognition moieties too short for simultaneous binding (Liu et al., 2015). An alternative possibility is that the short linkers enable a single gp120 subunit to simultaneously engage multiple CAR molecules, each by a distinct recognition component. Additional studies will be required to unravel the mechanistic complexities underlying the relationships between CAR multispecificity and potency.

The trispecific CD4-MBL-R5Nt CAR reported herein has significant advantages over the previously favored bispecific CD4-MBL CAR, namely greater anti-HIV potency (Figure 4B) and absence of detectable HIV entry receptor activity for the CD4 moiety (Figure 5B). This construct is thus a favored candidate for advancing to preclinical studies in relevant murine and simian models. Such studies will help assess the *in vivo* benefits of the trispecific design, with the goal of evaluating its potential in the HIV cure agenda.

## Materials and Methods

### PBMCs and Cell Lines

Buffy coats from healthy donors were obtained with informed consent from the NIH Clinical Center Department of Transfusion Medicine under Protocol 99-CC-0168. Peripheral blood mononuclear cells (PBMCs) were isolated using a Ficoll-Hypaque grandient. Frozen aliquots were stored in Recovery Cell Culture Freezing Medium (Thermo), and when thawed, grown in an AIM-V-based media (Thermo), containing 5% human AB serum (Valley Biomedical) and recombinant human interleukin-2 (Chiron).

GP2-293 cells (Clontech), used for retroviral vector production, were cultured in DMEM supplemented with 10% fetal bovine serum, 4 mM L-glutamine, 100 U/ml penicillin and 100 μg/ml streptomycin and the cells were grown at 37°C with 5% CO_2_.

Chinese hamster ovary (CHO) cells and CHO cells stably expressing HIV Env (CHO-*env*) were grown in DMEM containing 10% FBS, 2 mM glutamine, 1% non-essential amino acids, and 25 mM HEPES buffer. In addition, 250 nM methotrexate (MTX) was added to the growth medium of CHO-*env* cells, which constitutively express the Env protein from HIV-1 isolate III_B_ (Pitts et al., 1991). All cell culture media contained 100 U/ml penicillin and 100 μg/ml streptomycin and the cells were grown at 37°C with 5% CO_2_.

Human osteosarcoma cells expressing CCR5 (HOS.CCR5) cells were obtained from the NIH AIDS Reagent Program and grown DMEM containing 10% heat-inactivated fetal bovine serum (FBS), 100 U/ml penicillin, 100 μg/ml streptomycin, and 1:100 dilution of GlutaMAX supplement. On the day of use, 1 μg/ml of puromycin was added. The cells were grown at 37°C with 5% CO_2_.

### Plasmids

The CAR-encoding retroviral constructs are all based on the pMSGV-1 gammaretroviral vector (Hughes et al., 2005). Each CAR constructs shares the same hinge region, transmembrane domain, cytoplasmic signaling domains of CD28 and CD3 zeta. The extracellular recognition domains are as indicated in the CAR's designations. For the multi-specific CARs the domains are listed N- to C-terminus. All of the CD4-containing CARs contain domains 1 and 2 of human CD4 (residues 1-208, including the 25 residue signal peptide). The negative control CAR (referred to as 139 CAR), contains a single-chain variable fragment (scFv) from human MAb 139 which is specific for a variant of the epidermal growth factor (EGF) receptor only found on glioma cells (Morgan et al., 2012). All sequences are codon optimized for human expression. Gene fragments were synthesized by GenScripts, and the final constructs were stitched together by overlap extension PCR. The CD4-R5Nt(Y/A) mutant was generated from the CD4-R5Nt car using site directed mutagenesis. All primers were obtained from Thermo. Schematic representations of the CAR constructs are shown in Figure 1, and the corresponding amino acid sequences are presented in Supplementary Figure 1.

### Retroviral Vector Production

The retroviral vectors carrying the gene encoding the various CARs were generated by transfection of GP2-293 cells as described previously (Kochenderfer et al., 2009). The day before the transfection the cells were plated out on poly-D (or L)-lysine coated plates in media without penicillin or streptomycin. After a brief wash antibiotic free media, Lipofectamine 2000 (Thermo) was used to transfect the cells with a 2:1 mixture of plasmids encoding the CAR and the RD114 envelope glycoprotein (Porter et al., 1996). The co-transfected cells were replenished with DMEM supplemented with 10% fetal bovine serum, 4 mM L-glutamine, 100 U/ml penicillin and 100 μg/ml streptomycin and incubated for 48 h at 37°C in 5% CO_2_. After the incubation the supernatant was collected and stored at −80°C in aliquots.

### Transduction of PBMCs With Retroviral Vectors Encoding the CARs

In preparation for transduction with the CAR-bearing retroviral constructs, the PBMCs were thawed, washed with growth media and resuspending at 2 × 10^6^ cells/ml in AIM-V containing 5% human AB serum, 300 IU/ml interleukin-2 (IL-2; Roche), and 50 ng/ml of OKT3, an anti-CD3 monoclonal antibody (Abcam). The 2 ml of the cell suspension was aliquoted into each well of a 24-well tissue culture coated plate and cultured in 5% CO_2_ at 37°C for 2 days. To prepare duplicate sets of transduction plates, RetroNectin reagent (TaKaRa Bio) was diluted to 10 μg/ml in PBS and added to wells of non-tissue culture-treated 6-well plates at 1.5 ml/well and stored overnight at 4°C. The following day, aliquots of the retrovirus containing supernatant was rapidly thawed and diluted 1:1 in AIM-V supplemented with 5% human AB serum. The RetroNectin reagent was removed from the 6-well plate and replaced with 2 ml/well of a 2% bovine serum albumin (BSA) solution in PBS and incubated at room temperature for 30 min. The wells were washed twice with PBS, 2 ml/well of the retroviral solution was added, and the plates were centrifuged at 2,000 × *g* for 2 h at 32°C.

The activated PBMCs were pooled from the wells of the 24-well plate and adjusted to 0.5 × 10^6^ cells/ml in AIM-V supplemented with 5% human AB serum and 300 IU/ml of IL-2. One retrovirus coated plate was used for the first transduction, and the duplicate plate was stored at 4°C until the following day for the second transduction. After aspirating the supernatant from the first retrovirus coated 6-well plate, 3 ml of the cell suspension was added (for a total of 1. 5 × 10^6^ cells/ml) and centrifuged for 10 min at 2,000 × *g* at 32°C. The plate was incubated for 24 h 37°C in 5% CO_2_. The next day, the cells were pipetted up and down to gently dislodge them from the wells and transferred to the duplicate retrovirus coated plate (prepared as described above). The cells were incubated at 37°C in 5% CO_2_ for up to 2 weeks in T25 flasks standing upright. For subculturing, the cells were adjusted to 0.5–1 × 10^6^ cells/ml in AIM-V supplemented with 5% human AB serum and 300 IU/ml of IL-2 about every 3 days.

### Analysis of CAR Expression by Flow Cytometry

Transduced cells were monitored for CAR expression starting at 3 days after the second transduction. Approximately 1 × 10^6^ cells were removed from the culture and washed with 2 ml fluorescence-activated cell sorter (FACS) buffer (0.4% BSA in PBS) and resuspended in 100 ul of antibody solution diluted in FACS buffer (0.4% BSA in PBS). The antibody solution included anti-CD3-PerCP-Cy5.5 (BD Pharm), anti-CD4-APC-Cy7 (BD Pharm), anti-CD8-PE-Cy7 or anti-CD8-Alexa Fluor 700 (both from BD Pharm), and Aqua fluorescent reactive dye (LifeTech). The cells were incubated in the antibody solution at room temperature for 30 min, then washed with 2 ml FACS buffer. The cells were resuspended in 250 μl 3% paraformaldehyde diluted in PBS. The instruments used for flow cytometry acquisition were either BD FACSCalibur or FACSCanto II (BD Biosciences), and data analysis was performed using FlowJo (Treestar).

### Env-Specific Activation of CAR T Cells

CHO or CHO-*env* cells were seeded into the wells of a 12-well tissue culture-treated plate at 0.3 × 10^6^ cells/wells and grown overnight at 37°C in 5% CO_2_. The following day the media was aspirated and 1 × 10^6^ CAR T cells were added in 2 ml volume of AIM-V supplemented with 5% human AB serum to each well. Each well also received 5 μg/ml Brefeldin (BioLegend), 2 μM Monensin (BioLegend), and anti-CD107a (LAMP-1)-Brilliant Violet 785 (BioLegend). The plate was centrifuged at 1000 rpm for 2–3 min and incubated for 6 h at 37°C in 5% CO_2_. After the incubation, the cells were pipetted up and down to gently resuspend the CAR T cells and collected into FACS tubes. The cells were washed with 2 ml FACS buffer and resuspended in 100 μl of the antibody mixture described previously for analysis of CAR expression. After a 30-min incubation, the cells were washed with 2 ml FACS buffer, and incubated in 200 μl Cytofix solution (BD Scientific) and incubated at room temperature for 20 min. The cells were washed with 2 ml Perm/Wash buffer (BD Scientific) then resuspended in 100 μl anti-interferon gamma-PE (BioLegend) diluted in Perm/Wash buffer. After a 15 min incubation the cells were washed with 2 ml Perm/Wash buffer, and resuspended in 250 μl 3% paraformaldehyde diluted in PBS. Data was acquired using either BD FACSCalibur or FACSCanto II (BD Sciences), and data analysis was performed using FlowJo (Treestar).

### Inhibition of Spreading of Primary HIV-1 Infection by CAR-T Cells

To prepare targets cells for the infection, an aliquot of autologous PBMCs were rapidly thawed, washed and resuspended at 2 × 10^6^/ml in AIM-V containing 5% human AB serum, 300IU/ml IL-2, and 5 μg/ml phytohemagglutinin (PHA; Sigma). The cells were added to wells of a 24-well tissue culture-treated plate at 2 ml/well and incubated at 37°C in 5% CO_2_ for 1 day. Subsequently, the cells were pooled, washed in PBS and resuspended at a density of 0.5–1 × 10^6^ cells/ml in AIM-V supplemented with 5% human AB serum and 300 IU/ml of IL-2. The cells were incubated in an upright T25 flask at 37°C in 5% CO_2_ for 3 days.

In preparation for infection with HIV, 10 million cells the cells were washed with PBS and resuspended in AIM-V complemented with 5% human AB serum and mixed with 1 ml volume of primary HIV-1 isolate stock (p24 titer of 50–150 ng/ml). The final volume is 4 ml including 30 IU/ml of IL-2 in an upright T25 flask. The cells were incubated at 37°C in 5% CO_2_ for 1 day. The following day the infected “target” cells were washed three times with an excess volume of PBS and resuspended at 1 × 10^6^/ml in AIM-V containing 5% human AB serum, 30 IU/ml IL-2. The CAR-expressing “effector” cells (see “Transduction of PBMCs with retroviral vectors encoding the CARs” section), were washed with PBS and also resuspended at 1 × 10^6^/ml in AIM-V containing 5% human AB serum, 30 IU/ml IL-2. The effector cells were serially diluted in 1:5 dilutions in the wells of a 96-well round bottom tissue culture-treated plate, so that the most concentrated well contained 100,000 cells in 100 μl volume. Subsequently, 100 μl of target cells were added to each well. The resulting co-cultures contain effector-to-target ratios of 1:1, 0.2:1, 0.04:1, and 0.008:1 in a 200 μl volume. The plate was incubated at 37°C in 5% CO_2_ for up to 19 days. At 2 or 3 day intervals 180 ul of the supernatant was aspirated and saved for analysis. To monitor the level of infection in the wells, the p24 content of the supernatant was determined by HIV p24 AlphaLISA Detection Kit (Perkin Elmer). The signals were evaluated using an EnSpire Multimode Plate Reader (Perkin Elmer).

### Susceptibility of CAR-Expressing HOS.CCR5 Cells to Pseudovirus Entry

HOS cells expressing CCR5 were seeded out in DMEM containing 10% heat-inactivated fetal bovine serum (FBS), and 1:100 dilution of GlutaMAX supplement to be confluent the following day. On the following day, the media was replaced with fresh media and the cells were transfected with the various CAR-expression constructs described above using Lipofectamine 2000 transfection reagent. After transfection the media was replaced with DMEM containing 10% heat-inactivated fetal bovine serum (FBS), 100 U/ml penicillin, 100 μg/ml streptomycin, and 1:100 dilution of GlutaMAX supplement, as well as 1 μg /ml of puromycin and incubated at 37°C in 5% CO_2_ for 48 h. After the incubation the cells were harvested from the transfection plates using CellStripper Dissociation Reagent (Thermo) and washed. About 0.5 × 10^6^ cells were removed and stained with anti-CD4-APC-Cy7 (BD Pharm) and Aqua fluorescent reactive dye (LifeTech) and analyzed on a flow cytometer to monitor expression levels of the CD4-containing CARs. The cells were added to wells of a 96-well flat bottom plate at 3.5 × 10^6^ cells/well. Pseudovirus stocks with Env either from Ba-L or YU2 primary isolates and carrying a luciferase (Luc) gene were diluted 1:5 or 1:25 and added to the cells. The wells also contained 20 μg/ml DEAE-dextran. The plates were incubated at 37°C in 5% CO_2_ for 48 h. After the incubation the plates were centrifuged at 1,500 rpm for 5 min and the media was aspirated. The cells were evaluated for luciferase production using the Bright-Glo Luciferase Assay System (Promega), and the plates were read on an Ensight Multimode Plate Reader (Perkin Elmer).

### Biosecurity and Institutional Safety Procedures

All experiments and samples with infectious HIV were performed and maintained in the Biosafety Level 3 laboratory with Biosafety Level 2 practices, in accordance with Building 33 NIAID requirements. All other procedures were conducted according to NIH/NIAID standard biosecurity and safety regulations.

## Data Availability Statement

All datasets generated for this study are included in the article/Supplementary Material.

## Author Contributions

AH contributed to design of CAR constructs and experiments, conduct of experimental work including production of CAR constructs and retroviral vectors, HIV/CAR-T cell coculture assays, pseudovirus entry assays, analysis of data, and drafting of manuscript at multiple stages. DD contributed to conduct of experimental work including production of CAR constructs and retroviral vectors and analysis of data. DE contributed to conduct of experiments including production of CAR constructs and retroviral vectors and analysis of data. VB contributed to conduct of experimental work including production of CAR constructs and retroviral vectors, and HIV/CAR-T cell coculture assays and analysis of data. AS contributed to experimental work including production plasmids and corresponding soluble proteins and analysis of data. EB oversaw all aspects of CAR design, characterization of CAR expression and function in coculture assays, obtained funding, and contributed to drafting of this manuscript at multiple stages.

## Conflict of Interest

EB is co-inventor on patent applications for CD4-based bispecific CARs, owned by the National Institutes of Health. The remaining authors declare that the research was conducted in the absence of any commercial or financial relationships that could be construed as a potential conflict of interest.
