# Supplementary material for: A Trispecific Anti-HIV Chimeric Antigen Receptor Containing the CCR5 N-Terminal Region
Source: Front Cell Infect Microbiol. 2020 May 25;10:242. doi: 10.3389/fcimb.2020.00242 (PMC7261873; doi:10.3389/fcimb.2020.00242)
Supplement: Supplementary file 1 [file Data_Sheet_1.pdf]

Supplementary Figure 1

139 CAR:

139 scFv (signal peptide underlined)
Linker sequence
CD28 segment (transmembrane domain underlined)
CD3-zeta segment
P2A self-cleaving peptide (followed by the gene for the ZsGreen reporter, which had no effect on CAR expression or function)

MVLLVTSLLLCELPHPAFLLIPDIQMTQSPSSLSASVGDRVTITCRASQGIRNNLAWYQQKPGK
APKRLIYAASNLQSGVPSRFTGSGSGTEFTLIVSSLQPEDFATYYCLQHHSYPLTSGGGTKVEI
KRTGSTSGSGKPGSGEGSEVQVLESGGGLVQPGGSLRLSCAASGFTFSSYAMSWVRQAPGKGLE
WVSAISGSGGSTNYADSVKGRFTISRDNSKNTLYLQMNSLRAEDTAVYYCAGSSGWSEYWGQGT
LVTVSSAAAIEVMYPPPYLDNEKSNGTIIHVKGKHLCPSPLFPGPSKPFWVLVVVGGVLACYSL
LVTVAFIIFWVRSKRSRLLHSDYMNMTPRRPGPTRKHYQPYAPPRDFAAYRSRVKFSRSADAPA
YQQGQNQLYNELNLGRREEYDVLDKRRGRDPEMGGKPRRKNPQEGLYNELQKDKMAEAYSEIGM
KGERRRGKGHDGLYQGLSTATKDTYDALHMQALPPRATNFSLLKQAGDVEENPG

CD4 CAR:

CD4, D1D2 (signal peptide underlined)
Linker sequence
CD28 segment (transmembrane domain underlined)
CD3-zeta segment

MNRGVPFRHLLLVLQLALLPAATQGKKVVLGKKGDTVELTCTASQKKSIQFHWKNSNQIKILGN
QGSFLTKGPSKLNDRADSRRSLWDQGNFPLIIKNLKIEDSDTYICEVEDQKEEVQLLVFGLTAN
SDTHLLQGQSLTLTLESPPGSSPSVQCRSPRGKNIQGGKTLSVSQLELQDSGTWTCTVLQNQKK
VEFKIDIVVLAFQKASAAAIEVMYPPPYLDNEKSNGTIIHVKGKHLCPSPLFPGPSKPFWVLVV
VGGVLACYSLLVTVAFIIFWVRSKRSRLLHSDYMNMTPRRPGPTRKHYQPYAPPRDFAAYRSRV
KFSRSADAPAYQQGQNQLYNELNLGRREEYDVLDKRRGRDPEMGGKPRRKNPQEGLYNELQKDK
MAEAYSEIGMKGERRRGKGHDGLYQGLSTATKDTYDALHMQALPPR

CD4-MBL CAR:

CD4, D1D2 (signal peptide underlined)
Linker sequence
Carbohydrate recognition of domain of mannose-binding lectin (MBL)
CD28 segment (transmembrane domain underlined)
CD3-zeta segment
P2A self-cleaving peptide (followed by the gene for the ZsGreen reporter, which had no effect on CAR expression or function)

MNRGVPFRHLLLVLQLALLPAATQGKKVVLGKKGDTVELTCTASQKKSIQFHWKNSNQIKILGN
QGSFLTKGPSKLNDRADSRRSLWDQGNFPLIIKNLKIEDSDTYICEVEDQKEEVQLLVFGLTAN
SDTHLLQGQSLTLTLESPPGSSPSVQCRSPRGKNIQGGKTLSVSQLELQDSGTWTCTVLQNQKK
VEFKIDIVVLAFQKASGGGGSKQVGNKFFLTNGEIMTFEKVKALCVKFQASVATPRNAAENGAI
QNLIKEEAFLGITDEKTEGQFVDLTGNRLTYTNWNEGEPNNAGSDEDCVLLLKNGQWNDVPCST
SHLAVCEFPIAAAIEVMYPPPYLDNEKSNGTIIHVKGKHLCPSPLFPGPSKPFWVLVVVGGVLA
CYSLLVTVAFIIFWVRSKRSRLLHSDYMNMTPRRPGPTRKHYQPYAPPRDFAAYRSRVKFSRSA
DAPAYQQGQNQLYNELNLGRREEYDVLDKRRGRDPEMGGKPRRKNPQEGLYNELQKDKMAEAYS
EIGMKGERRRGKGHDGLYQGLSTATKDTYDALHMQALPPRATNFSLLKQAGDVEENPG

CD4-R5Nt CAR:

CD4, D1D2 (signal peptide underlined)
Linker sequence
CCR5 Nt segment
CD28 segment (transmembrane domain underlined)
CD3-zeta segment

MVRGVPFRHLLLVLQLALLPAATQGKKVVLGKKGDTVELTCTASQKKSIQFHWKNSNQIKILGN
QGSFLTKGPSKLNDRADSRRSLWDQGNFPLIIKNLKIEDSDTYICEVEDQKEEVQLLVFGLTAN
SDTHLLQGQSLTLTLESPPGSSPSVQCRSPRGKNIQGGKTLSVSQLELQDSGTWTCTVLQNQKK
VEFKIDIVVLAFQKASGGGGSGGGGSDYQVSSPIYDINYYTSEPSQKINVKAAAIEVMYPPPYL
DNEKSNGTIIHVKGKHLCPSPLFPGPSKPFWVLVVVGGVLACYSLLVTVAFIIFWVRSKRSRLL
HSDYMNMTPRRPGPTRKHYQPYAPPRDFAAYRSRVKFSRSADAPAYQQGQNQLYNELNLGRREE
YDVLDKRRGRDPEMGGKPRRKNPQEGLYNELQKDKMAEAYSEIGMKGERRRGKGHDGLYQGLST
ATKDTYDALHMQALPPR

CD4-R5Nt(Y/A) CAR:

CD4, D1D2 (signal peptide underlined)
Linker sequence
CCR5 Nt segment with 4 Y-to-A substitutions
CD28 segment (transmembrane domain underlined)
CD3-zeta segment

MVRGVPFRHLLLVLQLALLPAATQGKKVVLGKKGDTVELTCTASQKKSIQFHWKNSNQIKILGN
QGSFLTKGPSKLNDRADSRRSLWDQGNFPLIIKNLKIEDSDTYICEVEDQKEEVQLLVFGLTAN
SDTHLLQGQSLTLTLESPPGSSPSVQCRSPRGKNIQGGKTLSVSQLELQDSGTWTCTVLQNQKK
VEFKIDIVVLAFQKASGGGGSGGGGSDAQVSSPIADINAATSEPSQKINVKAAAIEVMYPPPYL
DNEKSNGTIIHVKGKHLCPSPLFPGPSKPFWVLVVVGGVLACYSLLVTVAFIIFWVRSKRSRLL
HSDYMNMTPRRPGPTRKHYQPYAPPRDFAAYRSRVKFSRSADAPAYQQGQNQLYNELNLGRREE
YDVLDKRRGRDPEMGGKPRRKNPQEGLYNELQKDKMAEAYSEIGMKGERRRGKGHDGLYQGLST
ATKDTYDALHMQALPPR

CD4-MBL-R5Nt CAR:

CD4, D1D2 (signal peptide underlined)
Linker sequence
Carbohydrate recognition of domain of mannose-binding lectin (MBL)
CCR5 Nt segment
CD28 segment (transmembrane domain underlined)
CD3-zeta segment

MNRGVPFRHLLLVLQLALLPAATQGKKVVLGKKGDTVELTCTASQKKSIQFHWKNSNQIKILGN
QGSFLTKGPSKLNDRADSRRSLWDQGNFPLIIKNLKIEDSDTYICEVEDQKEEVQLLVFGLTAN
SDTHLLQGQSLTLTLESPPGSSPSVQCRSPRGKNIQGGKTLSVSQLELQDSGTWTCTVLQNQKK
VEFKIDIVVLAFQKASGGGGSKQVGNKFFLTNGEIMTFEKVKALCVKFQASVATPRNAAENGAI
QNLIKEEAFLGITDEKTEGQFVDLTGNRLTYTNWNEGEPNNAGSDEDCVLLLKNGQWNDVPCST
SHLAVCEFPIGGGGSDYQVSSPIYDINYYTSEPSQKINVKAAAIEVMYPPPYLDNEKSNGTIIH
VKGKHLCPSPLFPGPSKPFWVLVVVGGVLACYSLLVTVAFIIFWVRSKRSRLLHSDYMNMTPRR
PGPTRKHYQPYAPPRDFAAYRSRVKFSRSADAPAYQQGQNQLYNELNLGRREEYDVLDKRRGRD
PEMGGKPRRKNPQEGLYNELQKDKMAEAYSEIGMKGERRRGKGHDGLYQGLSTATKDTYDALHM
QALPPR
